# A Rare but Fascinating Disorder: Case Collection of Patients with Schnitzler Syndrome

**DOI:** 10.1155/2018/7041576

**Published:** 2018-03-08

**Authors:** Maaman Bashir, Brittany Bettendorf, Richard Hariman

**Affiliations:** ^1^Division of Rheumatology, Department of Medicine, Medical College of Wisconsin, Milwaukee, WI 53226, USA; ^2^Division of Rheumatology, Department of Medicine, University of Iowa, Iowa City, IA 52242, USA

## Abstract

**Background:**

Schnitzler syndrome is a rare disorder characterized by a chronic urticarial rash and monoclonal gammopathy (IgM in more than 90% of the cases). It is difficult to distinguish from other neutrophilic urticarial dermatoses, and diagnosis is based on the Strasbourg criteria. Interleukin-1 is considered the key mediator, and interleukin-1 inhibitors are considered first line treatment. Here, we present two cases of Schnitzler syndrome, both successfully treated with anakinra.

**Objectives:**

To increase awareness regarding clinical presentation, diagnosis, and treatment of this rare disorder.

**Cases:**

We describe the clinical features and disease course of two patients with Schnitzler syndrome, diagnosed using the Strasbourg criteria. Both were treated with anakinra with remarkable response to therapy.

**Conclusion:**

Schnitzler syndrome is a rare and underdiagnosed disorder. High suspicion should be maintained in patients with chronic urticaria-like dermatoses, intermittent fevers, and arthralgias. A serum protein electrophoresis and immunofixation should be performed in these patients. The diagnosis is important to recognize as Schnitzler syndrome is associated with malignancy. A lymphoproliferative disorder develops in about 20% of patients at an average of 7.6 years after onset of symptoms. Thus, patients warrant long-term follow-up. IL-1 inhibitors are extremely effective in relieving symptoms and are considered first line therapy.

## 1. Introduction

Schnitzler syndrome is a rare disorder in the family of neutrophilic urticarial dermatoses with fewer than 300 reported cases [1]. The syndrome can often be difficult to recognize, and the diagnosis can easily be confused with one of the other NUD counterparts, including adult-onset Still's disease, lupus erythematosus, and cryopyrin-associated periodic syndromes [2]. Initially described in 1972 by the French dermatologist Liliane Schnitzler [3, 4], the disorder is diagnosed when patients meet the Strasbourg criteria. This includes two obligate criteria: recurrent, nonpruritic urticaria and monoclonal gammopathy (IgM kappa light chain in >90%) [1]. At least two of the following minor criteria are also required: recurrent fever, objective findings of abnormal bone remodeling with or without bone pain (assessed by bone scintigraphy, MRI, or elevation of bone alkaline phosphatase), neutrophilic dermal infiltrate on skin biopsy, and elevated CRP and/or leukocytosis (CRP > 30 mg/L and/or neutrophils > 10,000/mm^3^) [4]. The diagnosis is considered definite if the two obligate criteria and at least two minor criteria are met if the patient has IgM monoclonal gammopathy. The two obligate criteria and three minor criteria are required if there is IgG monoclonal gammopathy.

Skin biopsy from patients with the disorder is categorized as a neutrophilic urticarial dermatosis with histopathology demonstrating perivascular and interstitial neutrophilic inflammation with leukocytoclasia but without leukocytoclastic vasculitis [2, 5]. The pathogenesis of the disease remains unknown although it is thought to be autoinflammatory [2]. The disorder is best treated with medications that inhibit IL-1 such as anakinra, canakinumab, and rilonacept, but additional medications of symptomatic benefit have been identified including corticosteroids, rituximab, and cyclophosphamide [2]. A small case series also reported effectiveness of the anti-IL-6 receptor monoclonal antibody, tocilizumab, in patients who did not tolerate IL-1 inhibitors [6].

The diagnosis is important to recognize as Schnitzler syndrome is associated with malignancy. A lymphoproliferative disorder develops in about 20% of patients [2] at an average of 7.6 years after onset of symptoms and signs of Schnitzler syndrome [2].

## 2. Case 1

### 2.1. Presenting Concerns

The patient is a 56-year-old female with a past medical history of hypertension, hyperlipidemia, and ulcerative colitis who presented with complaints of joint pains for over 30 years. Her symptoms started with pain in the back radiating down the leg which later progressed to involve her shoulders, arms, wrists, and legs. She also described occasional swelling in her ankles, nonrefreshing sleep, and joint stiffness.

Eight years before presenting to our clinic, she was given a diagnosis of chronic Lyme disease based on non-CDC-approved testing and received IV ceftriaxone. However, repeat Lyme testing with IgG and IgM antibody by our facility was negative. MRI of her tibia and fibula two years prior to presenting in our clinic had revealed marrow edema, but a biopsy of her bone marrow was normal.

Upon initial presentation to our clinic, she was felt to have inflammatory arthritis and was treated with prednisone taper starting at 40 mg daily and weaned off slowly over several months. This helped her joint pain but did not resolve her symptoms completely. She was then trialed on treatment with hydroxychloroquine, methotrexate, and pregabalin without significant improvement.

### 2.2. Clinical Findings

At the time of diagnosis, exam revealed raised erythematous papules and plaques consistent with urticaria. They were distributed over the neck, upper back, and some on the arms. There were no signs of palpable purpura or necrosis. On joint exam, she had tenderness and swelling on palpation of bilateral 3rd PIPs. There was also tenderness in both shoulders, elbows, forearms, pretibial regions, and ankles without any swelling, erythema, or warmth. There was no evidence of synovitis or dactylitis in the feet.

### 2.3. Diagnostic Focus and Assessment

A detailed workup was performed (including ANA, ENA panel, RF, ANCA, serum protein electrophoresis and immunofixation, Lyme serology, muscle enzymes, ESR, CRP, ferritin, and cryoglobulins). She was found to have elevated inflammatory markers, with ESR 48 mm/hr, CRP 4.3 mg/dL, and ferritin 320.2 ng/mL. The electrophoresis and immunofixation revealed an IgM kappa monoclonal gammopathy. The rest of the blood work was negative. A bone survey of all long bones and a bone scan were also performed which showed diffuse osteitis and remodeling of long bones. Based on the urticarial rash, IgM kappa monoclonal gammopathy, abnormal bone remodeling, and elevated acute phase reactants, she fulfilled the Strasbourg criteria for diagnosis of Schnitzler syndrome.

### 2.4. Therapeutic Focus and Assessment

She was started on anakinra 100 mg subcutaneous daily. The patient also tested quantiferon positive and was started on isoniazid and vitamin B-6 simultaneously for treatment of latent tuberculosis.

### 2.5. Follow-Up and Outcome

The patient noticed significant improvement in her urticarial rash within days after initiating the anakinra. Shortly after that, she also had progressive decline in her musculoskeletal pain and gradually became symptom free. The improvement was also reflected in her inflammatory markers. Her CRP dropped from 4.3 mg/dL to 0.7 mg/dL, and ESR dropped from 48 mm/hr to 4 mm/hr.

## 3. Case 2

### 3.1. Presenting Concerns

The patient is a 44-year-old male with a past medical history of HIV and Burkitt's lymphoma who presented to clinic with daily nocturnal fevers and rash for 3 months. He also complained of bilateral ankle swelling and pain for 1 month. The fevers initially occurred once a week but gradually increased in frequency. The rash typically became more prominent with the fever but not always. He denied any other joint pains.

He was initially evaluated by dermatology, where a biopsy revealed perivascular and interstitial mixed dermal inflammation, including neutrophils. The dermatologist suggested a trial of prednisone 60 mg daily, dapsone, and colchicine for possible Sweet's syndrome. However, there was no improvement.

He denied any foreign travel in years and did not have a history of tick or unusual animal exposures. His HIV was appropriately treated, and he had a negligible viral load. He had completed therapy for Burkitt's lymphoma 1 year ago and was in complete remission.

### 3.2. Clinical Findings

On initial exam in our clinic, pink, blanching, tender, warm, nonpruritic wheals were seen on his shoulders, arms, chest, and back. He also had bilateral ankle synovitis.

### 3.3. Diagnostic Focus and Assessment

Labs revealed elevated inflammatory markers, including CRP (17 mg/dL), ESR (95 mm/hr), and ferritin (625 ng/mL). White blood cell count was elevated to 12,700/mm^3^. His ANA, RF, SS-A, SS-B, and ANCA were negative. Repeat skin biopsy of the left shoulder revealed superficial and deep perivascular interstitial mixed inflammation with scattered neutrophils. The electrophoresis and immunofixation revealed monoclonal bands with faint IgM lambda.

Based on his chronic urticarial rash, IgM lambda monoclonal gammopathy, recurrent fever, neutrophilic infiltrate on skin biopsy, elevated inflammatory markers, and leukocytosis, a diagnosis of Schnitzler syndrome was made.

### 3.4. Therapeutic Focus and Assessment

After an extensive literature search regarding the safety and efficacy of anakinra use in patients with HIV and Burkitt lymphoma, anakinra was initiated. Prednisone was tapered off over a 2-month period.

### 3.5. Follow-Up and Outcome

The patient's rash improved within hours of the first anakinra injection. He did not have any further fevers once anakinra was initiated. His arthritis resolved over 1-2 months. Marked improvement was also noted in his inflammatory markers; 1 month after beginning treatment, his CRP improved from 17 mg/dL to 0.2 mg/dL, and ESR improved from 95 mm/hr to 8 mm/hr. The patient has continued to do well on daily anakinra.

## 4. Discussion

Schnitzler syndrome is likely underrecognized with an average delay to diagnosis of 5-6 years [2, 7] because of the nonspecific nature of the presentation with intermittent fever and rash. Urticarial rash is often the first symptom to appear in Schnitzler syndrome [8]. The similarities between Schnitzler syndrome and adult-onset Still's disease (AOSD), including urticarial rash, fever, joint pain, and leukocytosis, can make the two disease entities difficult to distinguish from each other. However, there are a few unique features that can be helpful in differentiating between the two. AOSD often presents with an initial pharyngitis, which is absent in Schnitzler syndrome. Additionally, ferritin in AOSD is very high whereas it rarely exceeds 1200 ng/mL in Schnitzler syndrome. [4, 8, 9]. Of course, the diagnosis of Schnitzler syndrome also requires monoclonal IgM or IgG, further differentiating this from AOSD. Urticarial vasculitis can also mimic Schnitzler syndrome; however, with urticarial vasculitis, skin biopsy should reveal features of true vasculitis with fibrinoid necrosis of small vessel walls, which should not be present in Schnitzler syndrome. Additionally, patients with urticarial vasculitis often have complement consumption and anti-C1q antibodies not observed in patients with Schnitzler syndrome [4].

It is difficult to draw direct conclusions about pathogenesis of Schnitzler syndrome due to the small number of biopsy-proven patients with available direct immunofluorescence studies. However, it has been proposed that the skin lesions seen in Schnitzler syndrome may be triggered by deposition of IgM in the epidermis and at the dermoepidermal junction [7]. Mutations in the *NLRP3* gene (nucleotide-binding oligomerization domain leucine-rich repeats containing pyrin domain 3) have also been proposed to play a role. No germline *NLRP3* mutation has been reported; however, somatic mosaicism of *NLRP3* mutations in the myeloid lineage has been previously reported in 2 patients with Schnitzler syndrome [10]. It is possible that infections such as HIV and tuberculosis may also play a causative role in the development of Schnitzler syndrome, as both HIV [11] and tuberculosis [12] are known to activate the *NLRP3* inflammasome, which induces IL-1-beta production. Interestingly, both patients in the case scenarios above had one of these infections. Systemic overproduction of interleukin-1-beta in patients with Schnitzler syndrome is thought to result in a profound loss of anti-inflammatory Th17 cell functionalities [13], consistent with the well-described excellent response to IL-1 receptor blockade in patients with Schnitzler syndrome. The response to anakinra is so immediate and striking, that it has been proposed that response to anakinra be added as a diagnostic criterion [14].

There have been successful case reports and small clinical trials with other IL-1-blocking medications as well, including canakinumab [15–17] and rilonacept [18]. A recent placebo-controlled study involving 20 patients with Schnitzler syndrome had promising results. 7 days after treatment, significantly more patients (5/7) in the canakinumab group showed complete clinical response as compared to those in the placebo group (0/13), highlighting its potential as a treatment option for this disease [19]. A small number of patients have also achieved long-term remission with the use of anakinra [20, 21], and some authors have proposed that after 2 years of complete remission, treatment can be stopped to assess whether symptoms persist [4]. However, other authors have noted that symptoms always recur after treatment is stopped [22], with one small study of patients on canakinumab demonstrating a median time to relapse of 72 days after the last canakinumab dose [15]. Flares can take several days to resolve after IL-1 blockade is restarted.

The overall prognosis for the disease is dependent on whether the patient develops a lymphoproliferative disorder. Lymphoma or Waldenstrom disease occurs in 15–20% of patients [20, 23–25]. Other less frequently associated lymphoproliferative disorders include lymphoplasmacytic lymphoma, chronic lymphocytic leukemia, splenic marginal zone lymphoma, multiple myeloma, and marginal zone B-cell lymphoma [2, 8, 9].

Although Schnitzler syndrome requires the presence of monoclonal gammopathy to establish the diagnosis, the significance of the type of monoclonal protein is unclear. In the study by Sokumbi et al., 2 of 3 patients (66%) with IgG monoclonal gammopathy went on to develop malignancy, whereas 7 of 17 patients (41%) with IgM gammopathy developed malignancy. Because Schnitzler syndrome is so rare, there are no large case series studying this association. This may present an opportunity for future study as it would provide prognostic information to patients and clinicians.

Other diseases associated with Schnitzler syndrome include AA amyloidosis in untreated patients, sensorimotor neuropathy, severe anemia of chronic inflammation, and even hearing loss [8]. Proposed criteria for monitoring patients with Schnitzler syndrome include clinical evaluation, CBC, and CRP every 3 months. Monitoring of MGUS should be as usually recommended, based on its serum level (once yearly if under 10 g/L, twice yearly if less than 30 g/L, and every 3 months if more than 30 g/L) [4]. This monitoring should include CBC with differential, SPEP, creatinine, calcium (if mIgG), LDH, and urine protein [4]. Providers should remain vigilant for increases in the monoclonal Ig level or new lymphadenopathy which should prompt further appropriate testing such as bone marrow or lymph node biopsy. Once the patient is successfully treated, parameters should be monitored twice per year [4].
